# Change, Correlates, and Structure of Personality Across Adulthood

**DOI:** 10.1093/geroni/igab046.2154

**Published:** 2021-12-17

**Authors:** Olivia Atherton, Emorie Beck

## Abstract

Personality is both stable and changing across the lifespan. However, many questions remain about the factors that account for individual differences in change, the consequences of personality for life outcomes, and how best to assess personality at different points in the lifespan. First, Olivia Atherton will discuss research on the development of the Big Five personality traits from young adulthood to midlife with a sample of Mexican-origin individuals, as well as sociodemographic and cultural predictors of personality change in this population. Second, Bill Chopik uses data from 90 countries to examine the consistency of age differences in positive personality traits in the second half of life, from midlife to old age, as well as how cultural characteristics moderate the terminal decline in positive personality traits. Third, Emorie Beck will present research demonstrating that personality traits from the Big Five to beyond are robustly associated with a number of key life events across countries, decades, sociodemographic moderators, and even when controlling for selection bias. Finally, Josh Jackson uses network psychometric techniques to examine coherence and differentiation among indicators of the Big Five from 14 to 85 in a large multinational sample, tracking age differences with consequences for the assessment of personality traits in older adulthood. We will conclude with a panel discussion of emerging issues in personality change, prediction, and assessment across adulthood, with each speaker providing unique experience and insight into the study of each area.

